# Our first review: an evaluation of effectiveness of root cause analysis recommendations in Hong Kong public hospitals

**DOI:** 10.1186/s12913-020-05356-6

**Published:** 2020-06-05

**Authors:** Yick Ting A Kwok, Alastair PY Mah, Katherine MC Pang

**Affiliations:** 1 Root Cause Analysis Review Workgroup, Hospital Authority, Kowloon, Hong Kong; 2 Quality and Safety Division, New Territories West Cluster, Hospital Authority, New Territories, Hong Kong; 3 Patient Safety and Risk Management Department, Quality and Safety Division, Head Office, Hospital Authority, Kowloon, Hong Kong; 4 School of Medicine, Faculty of Health, Deakin University, Geelong, Australia

**Keywords:** Root cause analysis, Incident investigation, Patient harm, Patient safety, Sentinel events, Hong Kong

## Abstract

**Background:**

To evaluate the effectiveness of root cause analysis (RCA) recommendations and propose possible ways to enhance its quality in Hong Kong public hospitals.

**Methods:**

A retrospective cross-sectional study was performed across 43 public hospitals and institutes in Hong Kong, reviewing RCA reports of all Sentinel Events and Serious Untoward Events within a two-year period. The incident nature, types of root causes and strengths of recommendations were analysed. The RCA recommendations were categorised as ‘strong’, ‘medium’ or ‘weak’ strengths utilizing the US’s Veteran Affairs National Center for Patient Safety action hierarchy.

**Results:**

A total of 214 reports from October 2016 to September 2018 were reviewed. These reports generated 504 root causes, averaging 2.4 per RCA report, and comprising 249 (49%) system, 233 (46%) staff behavioural and 22 (4%) patient factors. There were 760 recommendations identified in the RCA reports with an average of 3.6 per RCA. Of these, 18 (2%) recommendations were rated strong, 116 (15%) medium and 626 (82%) weak. Most recommendations were related to ‘training and education’ (466, 61%), ‘additional study/review’ (104, 14%) and ‘review/enhancement of policy/guideline’ (39, 5%).

**Conclusions:**

This study provided insights about the effectiveness of RCA recommendations across all public hospitals in Hong Kong. The results showed a high proportion of root causes were attributed to staff behavioural factors and most of the recommendations were weak. The reasons include the lack of training, tools and expertise, appropriateness of panel composition, and complicated processes in carrying out large scale improvements. The Review Team suggested conducting regular RCA training, adopting easy-to-use tools, enhancing panel composition with human factors expertise, promoting an organization-wide safety culture to staff and aggregating analysis of incidents as possible improvement actions.

## Strengths and limitations of this study

This study is the first study analyzing the root causes and evaluating the effectiveness of RCA recommendations in Hong Kong public hospitals.

This study used a robust methodology to analyse the root causes and recommendations of RCA reports.

This study highlighted the key factors contributing to the distribution of strengths of recommendations.

The study only reviewed the strengths of recommendations but not the work progresses, feasibilities and outcome measures.

There were no other local data available to benchmark the results.

## Background

Ensuring patient safety is always a major challenge for healthcare organisations. With the arousal of awareness in patient safety since the release of the Institute of Medicine report in 1999, initiatives in patient safety improvement have been launched in countries all over the world [1–4]. These patient safety initiatives may have arisen from recommendations identified through root cause analysis (RCA) which identified system vulnerabilities from past clinical incidents.

RCA is an investigation methodology commonly used in healthcare organisations aiming to learn from system failures of clinical incidents, and identify patient safety improvement initiatives leading to elimination or control of the risks leading to such events [5–8]. It analyses incidents retrospectively in a systematic approach under a just and fair environment. The products of an RCA are the contributory factors proximally leading to the incident, the root causes about latent factors from the system perspective and the action plans to prevent recurrence of similar incidents in the future [8–12].

Many hospitals use RCA to investigate adverse events, with an example being the U.S. Joint Commission requiring incidents to be systematically investigated [13]. Although organisations might have put vast resources, in terms of time and manpower, [10, 14] in conducting RCAs, the number of preventable clinical incidents did not seem to reduce as would have expected. Researchers speculated that this might be due to the low effectiveness of RCA recommendations, and studies evaluating the strengths of recommendations found a high proportion of weak recommendations were put forward [10, 14, 15]. The low RCA effectiveness was widely discussed and suggested to be caused by the limited focus of weak recommendations that tackle superficial issues, mainly attempting to modify human behaviour which is believed to be difficult to sustain and cannot effectively eliminate the inherent latent risks at the system level [10, 12, 16, 17]. The identified factors preventing the development of stronger recommendations include the flawed design and context of RCA, the lack of RCA training and expertise, poor leadership and the loose mechanisms in promoting a safety culture [10, 14, 16, 18, 19].

In Hong Kong, all 43 public hospitals and institutes are established under the Hong Kong Hospital Authority (HA). Sentinel Events (SEs) are mandatory reporting incidents for HA hospitals since 2007 [20]. The original definition of Sentinel Events was adopted from the Joint Commission on Accreditation of Healthcare Organisations in the US, and the list of SEs was referenced to the Australia’s Sentinel Events categories [21]. For these public hospitals, RCAs must be conducted to investigate the root causes of all SEs and provide corresponding recommendations. RCA panels have to be formed and comprise of members from the involved hospitals or institutes, technical experts with relevant clinical expertise, as well as representatives from the Hospital Authority Head Office (HAHO). In 2010, the HA further mandated performing RCA for events called Serious Untoward Events (SUEs), incidents that could have the potential for serious patient harm once occurred [20]. For SUEs, the RCA panel would comprise members nominated by the involved hospitals or institutes. An RCA report template is provided by HAHO to facilitate RCA panels to document their investigation results. The identities of involved staff and patients are not disclosed in any RCA reports to maintain confidentiality and reduce blame on individuals. According to the HA SE and SUE Policy, RCA reports have to be submitted to the HAHO for record within 8 weeks. A meeting between hospital representatives and the Quality and Safety Division of the HAHO is conducted every 6 months to follow up on the work progress of recommendations.

From October 2007 till September 2019, 445 SEs and 876 SUEs were reported in the 43 public hospitals and institutes of the HA [20, 22]. With RCA having been introduced in the HA for more than a decade, there has been doubts within the organisation about the effectiveness of RCA in promoting patient safety and its sustainability. Since no corresponding research study in evaluating the effectiveness of RCA recommendations has ever been conducted in Hong Kong, the objectives of this study were to evaluate the effectiveness of RCA recommendations and propose suggestions for improving the quality of incident investigations in Hong Kong.

## Methods

### Study design

A retrospective study of RCA reports of all SEs and SUEs reported in the HA across a 2 year period was undertaken by an RCA Reports Review Team (the Review Team). A recent timeframe was chosen for review to gain a cross-sectional understanding of the quality of contemporary reports and recommend areas for improvement. The incident nature, types of root causes and strengths of recommendations were analysed. The root causes (in some RCA reports they were referred to as ‘contributory factors’, and for simplification purposes these are also regarded as ‘root causes’ in this study) of the events were reviewed and categorised according to the Contributory Factors Classification Framework of the National Patient Safety Agency (NPSA) of the United Kingdom (UK) [23]. Other classification methods like the Human Factors Analysis Classification System (HFACS) and Conceptual Framework for the International Classification for Patient Safety of the World Health Organisation (WHO) were considered for analysis but were not chosen [13, 24]. The HFACS was not chosen because some of the root causes were difficult to classify in a trial analysis using some HA RCA reports due to the vague description of the root causes. The NPSA method is more comprehensive in each category with explanation than the WHO Framework, thus the former method is selected.

In evaluating the strengths of RCA recommendations, the action hierarchy of the United States of America’s Veteran Affairs National Center for Patient Safety was selected as this method is widely accepted internationally [12]. In our study, some local adaptations were made, for example, recommendation types from Hibbert’s study were included for easier classification of the action hierarchy [10]. In the evaluation, a ‘strong’, ‘medium’ or ‘weak’ strength is given to each recommendation by the Review Team. ‘Strong’ recommendations indicate the recommendations focus on system changes and are more sustainable while ‘weak’ recommendations are focusing on human behavioural change and are less effective in eliminating the risks. The ‘medium’ recommendations, e.g. a checklist, have elements of system improvement but still rely on human compliance to be effective.

The research was done without patient or public involvement.

### Data collection

All SE and SUE RCA reports conducted from October 2016 to September 2018 in the 43 public hospitals and institutes under the governance of the HA in Hong Kong were reviewed.

### The RCA reports review team

The Review Team comprised of 3 reviewers, in which 2 were the primary reviewers (YK and AM) and the remaining was the secondary reviewer (MP). The primary reviewers first analysed the root causes and recommendations from RCA reports independently. The classifications of root causes and recommendations were finalised if the two sets of results between the primary reviewers were the same. For results that had discrepancy, the primary reviewers would discuss the results for a consensus. Otherwise, the secondary reviewer would make the final decision on the classification if a mutually agreed decision between the primary reviewers could not be made. A descriptive analysis was then conducted to evaluate the types of root causes and strengths and types of recommendations.

### Statistical analysis

Cohen’s kappa coefficients, calculated by SPSS version 21, were used to measure the inter-rater reliability of the reviewers [25].

### Ethics approval and consent to participate

Approval from the Clinical Research Ethics Committee, Kowloon Central Cluster, which is the governing committee in research ethics for the Hospital Authority Head Office, was obtained.

## Results

A total of 214 SEs and SUEs from October 2016 to September 2018 were reported in the 43 public hospitals and institutes of the HA. The distribution of event types is listed in Table 1. Nearly two-thirds (137, 64%) of the events were SUEs related to ‘medication error which could have led to death or permanent harm’, followed by 29 (14%) SEs related to ‘retained instruments or other material after surgery / interventional procedure’. There was no SE related to ‘medication error resulting in major permanent loss of function or death’ in the review period.

**Table 1:** Distribution of Events by Event Types

	Type^a^	Event	No.	%
1	SE	Surgery / interventional procedure involving the wrong patient or body part	8	4%
2	SE	Retained instruments or other material after surgery / interventional procedure	29	14%
3	SE	ABO incompatibility blood transfusion	1	0%
4	SE	Medication error resulting in major permanent loss of function or death	0	0%
5	SE	Intravascular gas embolism resulting in death or neurological damage	2	1%
6	SE	Death of an inpatient from suicide (including home leave)	15	7%
7	SE	Maternal death or serious morbidity associated with labour or delivery	4	2%
8	SE	Infant discharged to wrong family or infant abduction	2	1%
9	SE	Other adverse events (excluding complications) resulting in permanent loss of function or death	1	0%
10	SUE	Medication error which could have led to death or permanent harm	137	64%
11	SUE	Patient misidentification which could have led to death or permanent harm	15	7%
**Total**			**214**	**100%**

^*a*^*SE* Sentinel Event; *SUE* Serious Untoward Event

These RCA reports generated 504 root causes, averaging 2.35 per RCA report. The distribution of root causes by types is listed in Table 2. Kappa values between the primary reviewers (YK and AM) was 0.637 and the primary reviewers and secondary reviewer separately was 0.765 (YK and MP) and 0.806 (AM and MP). The Kappa values indicated that there was a moderate to strong strength of agreement [25]. In the Task Factor of the NPSA method, the Review Team separately extracted one sub-factor related to policy or guideline adherence in Task Factor for analysis. This sub-factor, which is related to staff’s non-compliance with the existing policy or guidelines (the ‘violation’ root causes), was the most common factor amongst the root causes (152, 30%), followed by education and training (87, 17%) and staff factor (81, 16%). According to the nature of the 9 different factors in the NPSA method, the Review Team has grouped these factors into three major groups, namely the Patient Factors, the Staff Behavioural Factors and the System Factors. Since in error taxonomy, ‘violation’ is a type of human failure, the Review Team thus grouped the ‘violation’ factor into the Staff Behavioural Factors Group. The distribution of root causes according to our grouping was 49% (249/504), 46% (233/504) and 4% (22/504) in System Factors, Staff Behavioural Factors and Patient Factors respectively.

**Table 2:** Distribution of Root Causes by Factors

Factors by NPSA Contributory Factors Classification Framework	No.	%	Groups by the RCA Reports Review Team	No.	%
Patient	22	4%	Patient Factors	22	4%
Staff	81	16%	Staff Behavioural Factors	233	46%
Task - policy & guideline adherence (the ‘violation’ root causes)	152	30%			
Task – others	25	5%	System Factors	249	49%
Communication	58	12%			
Equipment	33	7%			
Work environment	17	3%			
Organisation	15	3%			
Education & training	87	17%			
Team	14	3%			
**Total**	**504**	**100%**	**Total**	**504**	**100%**

The Review Team identified 760 recommendations, with an average of 3.6 per RCA. The distribution of recommendations is listed in Table 3. Kappa values between the primary reviewers (YK and AM) was 0.647 and between the primary reviewers and secondary reviewer separately was 0.730 (YK and MP) and 0.842 (AM and MP). The Kappa values indicated that there was a good strength of agreement [25]. Three RCAs did not provide any recommendations, all were SEs related to ‘death of an inpatient from suicide (including home leave)’. For the 760 recommendations, 18 (2%), 116 (15%) and 626 (82%) of them had strong, medium and weak strengths respectively. Most of the recommendations were ‘training and education (include counselling)’ (466, 61%), ‘additional study/review’ (104, 14%) and ‘review/enhancement of policy/guideline’ (39, 5%). The first two categories of recommendations, which were categorised with weak strength, had contributed to 75% of all recommendations.

**Table 3:** Distribution of Recommendations by Strengths

Strength	Action	No.	%^a^
**Strong**	Architectural / physical plant changes	1	0%
	New devices with usability testing before purchasing	1	0%
	Engineering control, interlock, forcing functions	2	0%
	Simplify the process and remove unnecessary steps	3	0%
	Standardize on equipment or process or care maps	10	1%
	Tangible involvement and action by leadership	1	0%
	**Subtotal**	**18**	**2%**
**Medium**	Redundancy/back-up systems	3	0%
	Increase in staffing/decrease in workload	1	0%
	Software enhancements/modifications	15	2%
	Eliminate/reduce distractions	3	0%
	Checklist/cognitive aid	8	1%
	Eliminate look- and sound-alikes	1	0%
	Enhanced communication	4	1%
	Simulation training with refresher	6	1%
	Review/enhancement of policy/guideline/documentation/workflow	39	5%
	Review/re-evaluate use/appropriateness of equipment	5	1%
	Audit undertaken	25	3%
	Enhanced supervision	6	1%
	Implement a new team (frontline)	0	0%
	**Subtotal**	**116**	**15%**
**Weak**	Double checks	7	1%
	Warnings and labels	20	3%
	New procedure/memorandum/policy	29	4%
	Training and education (include counselling)	466	61%
	Additional study/analysis	104	14%
	**Subtotal**	**626**	**82%**
**Total (Strong + Medium + Weak)**		**760**	**100%**

^a^ Discrepancies in display results due to round-off

## Discussion

This study is the first research in Hong Kong in understanding the effectiveness of RCA since its mandatory use for investigating SEs in 2007. The results of this study, especially in the categorization of root causes and recommendations, can provide meaningful information for the Hong Kong in improving its quality in incident investigations and subsequent risk mitigation actions.

One of the major challenges for the RCA panels is to identify the system vulnerabilities contributing to the underlying latent failures of the organisation [14, 16]. According to the results by our grouping, about 46% of the root causes were identified to be related to staff behavioural factors, for example, violation, lack of vigilance and lapse of concentration. In some RCA reports, the Review Team noticed that only staff behavioural factors were identified and no other system factors had been identified. In fact, human failures like slips, lapse and mistakes are normal human behaviour and can be difficult to eliminate [26]. The identification of such root causes is only superficial and only demonstrates that humans are imperfect, but is not meaningful in solving the problem [14]. The large proportion of staff behavioural factors suggests that the RCA panels were not able to recognize different aspects of systems issues such as equipment and workflow design flaws, poor usability of system interface, work overload and inadequate safety culture [10, 14, 16, 18]. This observation in the HA can be explained by the reason that the last corporate-led RCA training in the HA was conducted in 2009 and most RCA members have not been formally trained in RCA investigations in the past decade given the lack of training opportunities. This is especially true for the clinical experts who are invited to join the RCA according to their respective clinical expertise. These experts, generally with more than 10 years of clinical experience, would have limited understanding in RCA, safety systems knowledge and improvement science, as these have only taught in the medical undergraduate curriculum in the recent decade in Hong Kong [27].

The ‘violation’ root causes likely demonstrates the misconception to the term ‘violation’ amongst RCA panels in our review. From a human factors perspective, ‘violations’ are deviations from safe operating practices, procedures, standards, or rules, and have to be deliberately performed by the staff [26]. The Review Team noticed many RCA reports concluded that the staff had violated the policy or guideline, which was contributed by the causal factors of staff ‘having forgotten to perform a checking step’ or ‘not being aware of the situation’. There was no further investigation on the reasons for ‘violation’ nor was evidence of the staff’s intention to deliberately violate the rules provided. This observation is of particular importance as such misconception in ‘violation’ might have led to an unfair judgement being made to the involved staff. It is important that all RCA panel members buy into the purpose and principles behind an RCA as an incident investigation method, and to identify what is wrong at the system level and promote learning and sharing [28]. Tools like the Culpability Decision Tree or its recently adapted version, Just Culture Guide by the UK National Health Service, would be helpful in facilitating RCA panels to differentiate violations from other factors causing the staff not to follow the policy or guidelines and bringing a non-blame culture to the organisation [29, 30].

At the HA public hospitals, ‘5 whys’ and fishbone diagram are the commonest tools used to identify root causes. Though easy to use, both techniques have their drawbacks and RCA panel members have to use these techniques with caution [31, 32]. Other incident investigation and human factors tools and techniques including fault tree analysis, cognitive walkthrough, task analysis, heuristic evaluation techniques and interview question bank, are very useful to facilitate investigation on the evaluation of workflow, equipment and user interface and support data analysis and should be considered in the investigation process [12, 33–38]. Currently, the HA RCA report template does not provide any of the above tools for the RCA panel to make reference. In many studies and organisations’ incident management guide, human factors considerations are key components in conducting a robust RCA while quality improvement expertise is vital to effective implementation and process monitoring of action plans. It is advised that RCA members should be formally trained in human factors to support incident investigation and identification of system issues, make use of different tools and techniques to facilitate the investigation and analysis, and understand improvement science to implement action plans effectively [10, 12, 14, 16, 28, 37–40].

The study results showed that most of the recommendations were weak. Observations of high proportion of weak RCA recommendations have been reported in other studies using similar methodologies [10, 14, 15]. The Review Team noticed that in most RCAs, when a root cause of staff behavioural factor was identified, the corresponding recommendations would generally be to share the incident in department meetings, re-educate the involved staff or enhance their awareness through one-off training. These are weak recommendations as they attempt to change the human behaviour but do not treat the underlying ‘why’ problems [10, 16]. The Review Team also noticed some recommendations were not clearly linked to the root causes. For example, in an incident of wrong dose of Gentamicin administration, one of the root causes was the unclear content of verbal communication among nurse, dispenser and pharmacist. The report did not mention how ‘unclear’ the communication was while the corresponding recommendations were to share the incident in a training forum and in the nurses’ meeting. The Review Team believed that the RCA panel in this incident should further elaborate how and why the communication had broken down and a specific enhancement in that particular communication process between the staff would be a more appropriate recommendation rather than solely sharing the incident with staff. Indeed, sharing of incidents and their findings is in the regular incident management process and should not be a specific recommendation [37, 38]. The recommendations written in RCA reports should be actions inducing systems changes [8]. If training is identified as a recommendation at last by the RCA panel like the example above, the training should explain the risks and consequences of not communicating effectively with other staff during a procedure, and teach the necessary knowledge and skills required to address this [41].

Other system factor root causes were also found to propose weak recommendations in our review. Studies suggested that the tendency of RCA panels to propose weak recommendations is generally caused by the lack of understanding in RCA, limited knowledge in the hierarchy of controls and human factors [10, 14, 16, 28]. The RCA panels might perceive the investigation to be restricted to within the department such that organisational issues at a broader level were ignored for discussion. Within such confines, the choices of recommendations implementable solely at the departmental level become limited and additional staff training or putting more reminders has been the prominent actions arising from RCAs [19]. Stronger recommendations are also known to be costly and require more attention and monitoring to complete [37, 38, 42]. Though the RCA Panels are nominated directly by Hospital Chief Executives in the HA, political considerations for driving organisational change are often required for stronger recommendations, and those affecting fundamental systems of the whole HA may be difficult to ignore. For example, all hospitals and institutes in the HA use the same electronic clinical management system for patient documentation and management. When an RCA panel has identified a loophole in the electronic clinical management system, instead of directly asking the Information Technology Department to make the corrective actions, the proposed actions have to go through a series of processes including a number of platforms at hospital and corporate levels for stakeholder consultation, seek approval from different hospital and organisational committees, and lastly conduct a series of feasibility tests by software technicians. This process usually takes months if not years to complete. During such processes, hospital staff may question the RCA panel’s capability as they believe that the panel has not identified the appropriate root causes, and some observers may still assign blame to the involved staff as this is more visible [16, 19].

In addition, staff may blame the RCA panel for introducing new or perceived unrealistic changes at the organisational level for one single adverse event which they believe to be contributed to by a staff’s mistake [17]. The organisation might also have difficulty in assessing the vulnerability of the system by one single event [16]. The RCAs investigating individual, similar incidents during this review were found to produce inconsistent recommendations. An example would be in incidents where known drugs that cause allergic reactions to patients were prescribed and administered. The causes were found to be due to the input of the allergen in the “free text” section of the electronic medication management system, and thus could not automatically check cross-sensitivity of the drugs to alert staff. However, in some reports the recommendations were to provide training or reinforce staff compliance in checking allergy histories, while some reports suggested converting the free text allergy entries into structured entries to enable drug allergy checking by the electronic system. These inconsistent recommendations may be a product of multiple RCAs being conducted for individual events that have similar causes rather than being collectively reviewed and identifying underlying themes. Such variations in recommendations could affect staff’s impression about the quality of RCA as a whole.

These anticipated difficulties and conflicts in proposing system modifying recommendations may encourage the shift to weaker recommendations to avoid the RCA panel being held responsible and taking up the role in the complicated consultation process [19]. Recommendations like training and education or ‘to explore the feasibility of implementing an action plan or revising the practice’ have become common, yet are considered weak as the feasibility in implementing the actions are not yet certain or concrete. Even though stronger recommendations leading to some system changes have been suggested, they are usually limited to the departmental level and do not reach the whole organisation. Similar incidents therefore still recur in the same facility.

The results provided insights to the Review Team in proposing suggestions to enhance RCA quality in public hospitals and other healthcare organisations. First, regular training for RCA panel members must be conducted, and systems thinking and human factors should be an essential component of the training. The correct concept of ‘violations’, or in general the human error model must be understood, and available RCA tools should be promoted and panelists trained in their application [43]. References of investigation tools can also be added to the existing HA RCA report template to facilitate ease of access and serves as a prompt for use. Promotion of no-blame culture to all hospital staff must also be carried out to encourage staff in focusing on system issues instead of blaming individuals [16]. Skills in writing RCA reports have to be developed with practice so that the root causes and recommendations can be written more specifically and enable a new reader to immediately understand the issues and solutions [18].

Second, members with human factors expertise should be invited to join RCA panels as it may help shift any focus on blaming individuals to identifying systems and design flaws. This expertise can also support the design of patient safety initiatives [10, 16, 43]. Inviting staff who understand the involved workflows can also help the RCA panel quickly understand the detailed nuances of the situation [12]. Training a core group of staff specialized in incident investigations within the organisation would be a possible solution to solve the RCA expertise and sustainability problems. This core group of “specialists” can retain their knowledge and skills in RCA and they would have more opportunities to participate in an RCA [18]. From a broader perspective, an independent institution as a third party, with positioned similarly to the National Aeronautics and Space Administration of the US or the Healthcare Safety Investigation Branch of the UK, [44, 45] in the territory could be developed and hospitals can draw on expertise from this institution to support incident investigations as an independent party. The advantages of these independent institutions include minimising internal conflict, promoting unbiasedness, accuracy and credibility of RCA findings, and separate incident investigations and learning from violation disciplinary actions [46].

Third, the HA must promote a safety culture to all staff, which includes understanding the goals of a RCA, and reduce the barriers of RCA panels in proposing stronger recommendations focusing on organisational changes [42]. Fourth, the HA should consider aggregating analysis of incidents to counter the inconsistencies that will inevitably arise from multiple RCAs investigating similar issues [6, 10, 16, 37, 38]. By implementing these suggestions, the Review Team believes that incident investigations and improvements arising from them will become more effective in the organisation. The root causes will be more focused on system defects and a higher proportion of strong or medium recommendations would be anticipated. Last but not least, Australia has reviewed its SE list in 2017 and launched a second version in December 2018 [47, 48]. With the current SE and SUE lists being implemented in Hong Kong for about a decade, the list should be reviewed to ensure it aligns with the goal of effectively monitoring and preventing serious adverse patient harm events.

This study has two limitations. First, the study only reviewed the strengths of recommendations but not the work progresses, feasibilities and outcomes of the recommendations. Due to the boundary of action hierarchy in categorizing recommendations of ‘additional study / analysis’ as weak, the Review Team believed that there will be more strong or medium actions taken when the recommendations are studied to be feasible. Though the HAHO follow up on the work progress of RCA recommendations every 6 months, the outcomes are not evaluated. The Review Team believes that incorporating measures of success in RCA Reports would be worthwhile to determine if the recommendations are effective. Second, this study is the first study in the HA to review RCA effectiveness. There was no other local data available for benchmarking. If improvements like conducting RCA training and improving the human factors expertise of RCA panel members are implemented, a follow-up study would be beneficial.

## Conclusion

This study provided insights to the HA about the effectiveness of RCA recommendations in the organisation. The results showed a high proportion of root causes were staff behavioural factors and most of the recommendations were weak. The reasons behind were the lack of training to RCA panel members especially in systems thinking and human factors knowledge, skills and tools, the lack of members with human factors expertise and staff who understand the clinical workflow in the RCA panel composition, and the complicated process in carrying out large scale improvements across the organisation. The Review Team suggested conducting regular RCA training, implementing easy-to-use RCA tools, inviting members with human factors expertise to the RCA panel, promoting a safety culture to staff in all public hospitals and aggregating analysis of incidents, to be possible actions adopted by the organisation’s management. A follow-up study could be considered after conducting the improvement actions. The HA should also consider reviewing the SE and SUE list to ensure serious adverse patient harm events are effectively monitored and prevented.

## Data Availability

The data that support the findings of this study are available from the Hong Kong Hospital Authority but restrictions apply to the availability of these data, which were used only for the current study, and so are not publicly available. Data are however available from the authors upon reasonable request and with permission of the Hospital Authority.

## Abbreviations

HA: Hospital Authority
HAHO: Hospital Authority Head Office
HFACS: Human Factors Analysis Classification System
NPSA: National Patient Safety Agency
RCA: Root Cause Analysis
SE: Sentinel Event
SUE: Serious Untoward Event
UK: United Kingdom
WHO: World Health Organisation
